# Biogenic Polyamines and Related Metabolites

**DOI:** 10.3390/biom12010014

**Published:** 2021-12-22

**Authors:** Alexander V. Ivanov, Alex R. Khomutov

**Affiliations:** Engelhardt Institute of Molecular Biology, Russian Academy of Sciences, 119991 Moscow, Russia

The specific regulation of cell metabolism is one of cornerstones of biochemistry. Numerous lines of evidence have been obtained showing that various pathologies are associated with changes in different metabolic pathways. Tumor cells, bacteria, fungi, and trypanosomatides are more often dependent on certain metabolites that normal cells, and targeting respective metabolic enzymes represents a promising strategy for the treatment of various types of diseases [1,2]. Viruses are even more dependent on host metabolites compared with tumor cells and bacteria. A variety of drugs that specifically affect virus enzymes are used as therapeutics. However, viruses and hosts compete for the same metabolites and there has only been a few attempts to tip the balance in favor of the host and restricting virus’s requirements for essential metabolites. Therefore, the analysis of interplay between pathologies and various metabolic pathways is of obvious scientific and practical importance.

Biogenic polyamines spermine (1,12-diamino-4,9-diazadodecane), spermidine (1,8-diamino-4-azaoctane) and their precursor putrescine (1,4-diaminobutane) represent natural compounds that are present in all cells at millimolar and submillimolar concentrations and are essential growth factors in eukaryotic cells [3]. However, polyamines and their metabolism have attributed much less attention than sugars, amino acids and other intermediates of the central carbon metabolism. Polyamines play really important roles in a wide range of biological processes, including DNA replication and transcription, RNA translation and frameshift, scavenging of reactive oxygen species, etc. [4,5]. As a result, the dysregulation of polyamine biosynthesis or catabolism can affect cell growth and differentiation and even result in cell death [6,7]. The aim this Special Issue is to promote research in a field of polyamines by bringing together papers from this field and from adjacent areas that are rarely considered in the context of polyamines.

Polyamine metabolism is maintained by several enzymes. Diamine putrescine, the precursor of spermidine and spermine, is produced from a non-proteinogenic amino acid ornithine of the urea cycle by ornithine decarboxylase (ODC), which is a rate-limiting polyamine biosynthetic enzyme. It is noteworthy that ornithine can also be formed by ornithine aminotransferase from glutamate/proline pathways. In bacteria, fungi and plants, putrescine can be also formed from arginine via agmatine by arginine decarboxylase (ADC) and agmatinase. The consequent attachment of the 3-aminopropyl group, derived from decarboxylated *S*-adenosylmethionine, to either side of putrescine results in spermidine and spermine, respectively. These polyamines are not only synthesized endogenously but are also supplied with food. The degradation of polyamines is achieved by a tandem of spermidine/spermine-*N*^1^-acetyl transferase (SSAT) and acetylpolyamine oxidase (PAOX) and also by spermine oxidase (SMOX). The expressions of ODC, SSAT and SMOX are tightly controlled in both positive and negative directions through a series of feedback circuits to maintain cellular functions without leading to malignant transformation of the cell. The key down-regulator of the polyamine pool is a small, short-living protein antizyme 1 (OAZ1), which is synthesized and works when polyamine concentration exceeds normal level. The biosynthesis of OAZ1 is stimulated by polyamines, which induces +1 ribosomal frameshift of OAZ1 mRNA needed to read-through the stop codon and to produce a full-length OAZ1 protein. To decrease the polyamine pool, OAZ1 binds to an ornithine decarboxylase (ODC), the rate-limiting enzyme of polyamine biosynthesis, and targets it for ubiquitin-independent degradation by the 26S proteasome. In this Special Issue, Kudriaeva et al. [8] demonstrate that spermine and spermidine boost the activity of proteasomes in a dose-dependent manner. This observation may be of interest for a better understanding of mechanisms of proteasome-mediated proteolysis and also for maintaining polyamine homeostasis in a cell.

*S*-Adenosylmethionine is one of the most abundant sources of methyl groups in a wide spectrum of trans-methylation reactions. About 10% of *S*-adenosylmethionine undergoes decarboxylation to serve as a source of a three-carbon unit of polyamine backbone, i.e., there is a direct crossroad of polyamine and *S*-adenosylmethionine metabolism. Small-molecule methylation is a part of the biosynthesis and modification of numerous cellular metabolites. RNA methylation regulates the translation, stability, and transport of mRNA, the quality of ribonucleoprotein assembly, splicing regulation, etc. Protein methylation increases a variety of protein amino acids, as it leads to the generation of non-canonical groups, thus regulating functional properties of proteins. The methylation of DNA affects chromatin condensation and plays a key role in the regulation of gene expression, genomic imprinting and carcinogenesis in eukaryotic cells. Human Dnmt3a and Dnmt3b methyltransferases mediate de novo methylation, thus forming the primary DNA methylation pattern. In this Special Issue, Khrabrova et al. [9] perform an analysis of the mutations occurring in the Dnmt3a catalytic domain and the effects of these mutations on the DNA binding and the formation of the transient covalent intermediate.

Spermine and spermidine, being aliphatic positively charged molecules, have many common functions realized presumably via ionic interactions with DNA, RNA, and proteins. However, various groups have reported that their functions do not fully overlap, as spermidine plays a predominant role in controlling cell differentiation [10,11]. This was shown using various approaches: by finding correlations between changes in the spermidine-to-spermine ratio with changes in cell (de)differentiation [12] and also by the use of functionally active, non-interconvertible methylated analogs of these polyamines [13,14]. One of the possible explanations for more profound roles of spermidine over spermine is its implication in a unique type of protein post-translational modification—hypusination, i.e., the conversion of lysine residue into *N*-ε-(4-amino-2-hydroxybutyl)-*L*-lysine. Hypusination is a two-step process of conjugating a spermidine fragment to a lysine residue of eIF5a (catalyzed by deoxyhypusine synthase, DHS) followed by the oxidation of one of the methylene groups (by deoxyhypusine hydrolase, DOHH). eIF5A hypusination is known to promote the translation of polyproline-rich stretches, and it also enhances translation termination by stimulating peptide release. By promoting translation, the hypusine modification of eIF5A provides a key link between polyamines and cell growth regulation [15]. A decrease in eIF5A hypusination is one of the most delayed consequences of the depletion of intracellular polyamine pools [16]. In line with this, inhibitors of both DHS and DOHH are considered as promising anticancer and antimicrobial agents. In this Special Issue, Wator et al. [17] reveal the molecular interaction of DHS with spermidine and other polyamines by solving the high-resolution crystal structures of the enzyme–ligand complexes. They reveal that spermidine is required for the formation of a functional tetramer and demonstrated mechanisms of high selectivity of the enzyme towards this polyamine.

A discovery of an association between high levels of polyamines and rapid proliferation of cancer cells significantly promoted research of polyamine biochemistry [18]. Therefore, the polyamine metabolism started to be considered as a target for anticancer drug development. Indeed, one of the major breakthroughs in the field is a successful treatment of child neuroblastoma using a standard combination of drugs together with the ODC inhibitor difluoromethylornithine (DFMO, Eflornithine) [19]. The use of the latter not only reduces rates of relapse but allows prolongation of life of patients who were previously resistant to the standard therapy lacking DFMO. However, as polyamines are not only synthesized in a cell but also actively taken up from the media, the development of inhibitors of polyamine uptake became of crucial importance. Now, this is one of the “hot points” of the field. In recent years, several transporters were unveiled, and their inhibitors were developed. In this Special Issue, Corral and Wallace demonstrate that import of polyamines is elevated in colon cancer cell lines [20]. Moreover, their data suggest the existence of separate transporters for spermidine and its precursor, putrescine. Since the inhibition of polyamine biosynthesis leads to an increase in their uptake, the newly developed inhibitors of polyamine uptake may further advance anticancer therapy.

Peculiarities of spermine and spermidine uptake machinery may be exploited for drug delivery or the induction of immune response [21]. Moreover, polyamine- and amine-catabolic enzymes themselves can be used as anticancer agents, as the degradation of aliphatic amines and polyamines is accompanied by the generation of hydrogen peroxide and toxic aldehydes. In the Special Issue, Magro et al. [22] review the possible applications of nanotechnologies for cancer treatment. Specifically, they show the data from theirs and other groups’ work demonstrating that nanoparticles efficiently deliver cytotoxic molecules to tumor cells and exemplify it by gold nanoparticles containing bovine serum amino-oxidases.

Maintenance of the required polyamine levels are of crucial importance for normal cell growth and differentiation. Therefore, modern polyamine research cannot exist without precise analytical methods allowing the determination of the polyamine content in cells and tissues. Different methods, including thin-layer chromatography (TLC), high-pressure liquid chromatography (HPLC) and using isotope dilution liquid chromatography with mass-spectrometric detection were developed and successfully used. In this Special Issue, Samarra et al. [23] published a LC-MS/MS stable isotope dilution method for the determination of polyamines and their tightly related metabolites in liquid biopsies. Sample extraction protocols and subsequent derivatization with dansyl chloride were optimized, and the suggested method might be of use for clinical studies.

Polyamines are critical not only for eukaryotes but also for bacteria and viruses. It has been clearly established that certain bacteria perturb polyamine homeostasis, and one of its features, the induction of SMOX expression by carcinogenic *Helicobacter pylori* [24] and *Bacteroides fragilis* [25] represents the mechanism of neoplastic transformation of gastric and colon tissues. Thus, SMOX is considered a target for pharmacological intervention. At the same time, the growth of bacteria and fungi might be blocked by inhibiting their unique pathway of polyamine biosynthesis, i.e., the production of putrescine via agmatine. Since mammals encode agmatinase in their genome, the compounds should target arginine decarboxylase. In a research paper of Hyvonen et al. within the Special Issue, novel highly active and selective nanomolar ADC inhibitors are described [26]. The effective inhibition of ADC was interpreted in terms of the similarity of the enzyme oxime and one of the reaction intermediates, as it was first demonstrated by X-ray for aspartate aminotransferase [27] and very recently for ODC [28]. The authors demonstrate the decreased growth of bacteria and fungi cells in the presence of hydroxylamine-analogue of agmatine. 

Much less is unknown about the interplay between polyamines and viral infections. Such studies were carried out in the 1970s and 1980s, with a huge gap since then. During the last decade, two groups, of Bryan Mounce and John Connor, advanced the field by showing that the inhibition of polyamine biosynthesis or eIF5a hypusination or activation of polyamine catabolism suppresses the replication of various DNA and RNA viruses. In an excellent review in the Special Issue, Firpo and Mounce summarize all these findings, as well as data on viral imprint on polyamine homeostasis in a host cell [29]. A very recent study from Mounce group showed that the compounds that target polyamine metabolism downregulate SARS-CoV-2 replication [30].

In recent decades, numerous examples have shown that the dysregulation of the polyamine metabolism is associated and, in many cases, contributes to the development of various types of cancers and autoimmune and metabolic diseases. The understanding of the cellular functions of polyamines at a molecular level and the discovery of novel approaches for the regulation of their metabolism will open new avenues for successful scientific interventions having also practical significance.

## References

[1] Stine Z.E., Schug Z.T., Salvino J.M., Dang C.V. (2021). Targeting cancer metabolism in the era of precision oncology. Nat. Rev. Drug Discov..

[2] Chen X., Qian Y., Wu S. (2015). The warburg effect: Evolving interpretations of an established concept. Free Radic. Biol. Med..

[3] Casero R.A., Marton L.J. (2007). Targeting polyamine metabolism and function in cancer and other hyperproliferative diseases. Nat. Rev. Drug Discov..

[4] Igarashi K., Kashiwagi K. (2019). The functional role of polyamines in eukaryotic cells. Int. J. Biochem. Cell Biol..

[5] Miller-Fleming L., Olin-Sandoval V., Campbell K., Ralser M. (2015). Remaining mysteries of molecular biology: The role of polyamines in the cell. J. Mol. Biol..

[6] Casero R.A., Murray Stewart T., Pegg A.E. (2018). Polyamine metabolism and cancer: Treatments, challenges and opportunities. Nat. Rev. Cancer.

[7] Handa A.K., Fatima T., Mattoo A.K. (2018). Polyamines: Bio-molecules with diverse functions in plant and human health and disease. Front. Chem..

[8] Kudriaeva A.A., Saratov G.A., Kaminskaya A.N., Vladimirov V.I., Barzilovich P.Y., Belogurov A.A. (2020). Polyamines counteract carbonate-driven proteasome stalling in alkaline conditions. Biomolecules.

[9] Khrabrova D.A., Loiko A.G., Tolkacheva A.A., Cherepanova N.A., Zvereva M.I., Kirsanova O.V., Gromova E.S. (2019). Functional analysis of dnmt3a DNA methyltransferase mutations reported in patients with acute myeloid leukemia. Biomolecules.

[10] Ishii I., Ikeguchi Y., Mano H., Wada M., Pegg A.E., Shirahata A. (2012). Polyamine metabolism is involved in adipogenesis of 3t3-l1 cells. Amino Acids.

[11] Ivanova O.N., Snezhkina A.V., Krasnov G.S., Valuev-Elliston V.T., Khomich O.A., Khomutov A.R., Keinanen T.A., Alhonen L., Bartosch B., Kudryavtseva A.V. (2018). Activation of polyamine catabolism by n(1),n(11)-diethylnorspermine in hepatic heparg cells induces dedifferentiation and mesenchymal-like phenotype. Cells.

[12] Alhonen L., Rasanen T.L., Sinervirta R., Parkkinen J.J., Korhonen V.P., Pietila M., Janne J. (2002). Polyamines are required for the initiation of rat liver regeneration. Biochem. J..

[13] Hyvonen M.T., Keinanen T.A., Khomutov M., Simonian A., Weisell J., Kochetkov S.N., Vepsalainen J., Alhonen L., Khomutov A.R. (2011). The use of novel c-methylated spermidine derivatives to investigate the regulation of polyamine metabolism. J. Med. Chem..

[14] Khomutov M., Hyvonen M.T., Simonian A., Formanovsky A.A., Mikhura I.V., Chizhov A.O., Kochetkov S.N., Alhonen L., Vepsalainen J., Keinanen T.A. (2019). Unforeseen possibilities to investigate the regulation of polyamine metabolism revealed by novel c-methylated spermine derivatives. J. Med. Chem..

[15] Park M.H., Wolff E.C. (2018). Hypusine, a polyamine-derived amino acid critical for eukaryotic translation. J. Biol. Chem..

[16] Chattopadhyay M.K., Park M.H., Tabor H. (2008). Hypusine modification for growth is the major function of spermidine in saccharomyces cerevisiae polyamine auxotrophs grown in limiting spermidine. Proc. Natl. Acad. Sci. USA.

[17] Wator E., Wilk P., Grudnik P. (2020). Half way to hypusine-structural basis for substrate recognition by human deoxyhypusine synthase. Biomolecules.

[18] Russell D., Snyder S.H. (1968). Amine synthesis in rapidly growing tissues: Ornithine decarboxylase activity in regenerating rat liver, chick embryo, and various tumors. Proc. Natl. Acad. Sci. USA.

[19] Evageliou N.F., Haber M., Vu A., Laetsch T.W., Murray J., Gamble L.D., Cheng N.C., Liu K., Reese M., Corrigan K.A. (2016). Polyamine antagonist therapies inhibit neuroblastoma initiation and progression. Clin. Cancer Res..

[20] Corral M., Wallace H.M. (2020). Upregulation of polyamine transport in human colorectal cancer cells. Biomolecules.

[21] Palmer A.J., Ghani R.A., Kaur N., Phanstiel O., Wallace H.M. (2009). A putrescine-anthracene conjugate: A paradigm for selective drug delivery. Biochem. J..

[22] Magro M., Venerando A., Macone A., Canettieri G., Agostinelli E., Vianello F. (2020). Nanotechnology-based strategies to develop new anticancer therapies. Biomolecules.

[23] Samarra I., Ramos-Molina B., Queipo-Ortuno M.I., Tinahones F.J., Arola L., Delpino-Rius A., Herrero P., Canela N. (2019). Gender-related differences on polyamine metabolome in liquid biopsies by a simple and sensitive two-step liquid-liquid extraction and lc-ms/ms. Biomolecules.

[24] Chaturvedi R., Asim M., Romero-Gallo J., Barry D.P., Hoge S., de Sablet T., Delgado A.G., Wroblewski L.E., Piazuelo M.B., Yan F. (2011). Spermine oxidase mediates the gastric cancer risk associated with helicobacter pylori caga. Gastroenterology.

[25] Goodwin A.C., Destefano Shields C.E., Wu S., Huso D.L., Wu X., Murray-Stewart T.R., Hacker-Prietz A., Rabizadeh S., Woster P.M., Sears C.L. (2011). Polyamine catabolism contributes to enterotoxigenic bacteroides fragilis-induced colon tumorigenesis. Proc. Natl. Acad. Sci. USA.

[26] Hyvonen M.T., Keinanen T.A., Nuraeva G.K., Yanvarev D.V., Khomutov M., Khurs E.N., Kochetkov S.N., Vepsalainen J., Zhgun A.A., Khomutov A.R. (2020). Hydroxylamine analogue of agmatine: Magic bullet for arginine decarboxylase. Biomolecules.

[27] Markovic-Housley Z., Schirmer T., Hohenester E., Khomutov A.R., Khomutov R.M., Karpeisky M.Y., Sandmeier E., Christen P., Jansonius J.N. (1996). Crystal structures and solution studies of oxime adducts of mitochondrial aspartate aminotransferase. Eur. J. Biochem..

[28] Zhou X.E., Suino-Powell K., Schultz C.R., Aleiwi B., Brunzelle J.S., Lamp J., Vega I.E., Ellsworth E., Bachmann A.S., Melcher K. (2021). Structural basis of binding and inhibition of ornithine decarboxylase by 1-amino-oxy-3-aminopropane. Biochem. J..

[29] Firpo M.R., Mounce B.C. (2020). Diverse functions of polyamines in virus infection. Biomolecules.

[30] Firpo M.R., Mastrodomenico V., Hawkins G.M., Prot M., Levillayer L., Gallagher T., Simon-Loriere E., Mounce B.C. (2021). Targeting polyamines inhibits coronavirus infection by reducing cellular attachment and entry. ACS Infect. Dis..

